# An observational field study of the cloacal microbiota in adult laying hens with and without access to an outdoor range

**DOI:** 10.1186/s42523-020-00044-6

**Published:** 2020-08-08

**Authors:** Janneke Schreuder, Francisca C. Velkers, Ruth J. Bouwstra, Nancy Beerens, J. Arjan Stegeman, Willem F. de Boer, P. van Hooft, Armin R. W. Elbers, Alex Bossers, Stephanie D. Jurburg

**Affiliations:** 1 Faculty of Veterinary Medicine, Department Population Health Sciences, Utrecht University, Yalelaan 7, 3584 CL Utrecht, The Netherlands; 2 GD Animal Health, Deventer, the Netherlands; 3 Department of Virology, Wageningen Bioveterinary Research, Lelystad, the Netherlands; 4 Wildlife Ecology and Conservation Group, Wageningen University & Research, Wageningen, the Netherlands; 5 Department of Bacteriology and Epidemiology, Wageningen Bioveterinary Research, Lelystad, the Netherlands; 6 Department of Infection Biology, Wageningen Bioveterinary Research, Lelystad, the Netherlands; 7 German Centre for Integrative Biodiversity Research (iDiv), Halle-Jena-Leipzig, Leipzig, Germany

**Keywords:** Microbiota, 16S rRNA, Poultry, Laying hen, Outdoor range

## Abstract

**Background:**

Laying hens with access to outdoor ranges are exposed to additional environmental factors and microorganisms, including potential pathogens. Differences in composition of the cloacal microbial community between indoor- and outdoor-housed layers may serve as an indicator for exposure to the outdoor environment, including its pathogens, and may yield insights into factors affecting the chickens’ microbiota community dynamics. However, little is known about the influence of outdoor housing on microbiota community composition in commercial layer flocks. We performed a cross-sectional field study to evaluate differences in the cloacal microbiota of indoor- vs outdoor-layers across farms.

Eight layer flocks (four indoor, four outdoor) from five commercial poultry farms were sampled. Indoor and outdoor flocks with the same rearing flock of origin, age, and breed were selected. In each flock, cloacal swabs were taken from ten layers, and microbiota were analysed with 16S rRNA gene amplicon sequencing.

**Results:**

Housing type (indoor vs outdoor), rearing farm, farm and poultry house within the farm all significantly contributed to bacterial community composition. Poultry house explained most of the variation (20.9%), while housing type only explained 0.2% of the variation in community composition. Bacterial diversity was higher in indoor-layers than in outdoor-layers, and indoor-layers also had more variation in their bacterial community composition. No phyla or genera were found to be differentially abundant between indoor and outdoor poultry houses. One amplicon sequence variant was exclusively present in outdoor-layers across all outdoor poultry houses, and was identified as *Dietzia maris*.

**Conclusions:**

This study shows that exposure to an outdoor environment is responsible for a relatively small proportion of the community variation in the microbiota of layers. The poultry house, farm, and rearing flock play a much greater role in determining the cloacal microbiota composition of adult laying hens. Overall, measuring differences in cloacal microbiota of layers as an indicator for the level of exposure to potential pathogens and biosecurity seems of limited practical use. To gain more insight into environmental drivers of the gut microbiota, future research should aim at investigating community composition of commercial layer flocks over time.

## Background

In recent years the demand for free-range poultry products has increased. Free-range housing for commercial laying chickens allows laying hens to access an outdoor range during the day, which is believed to benefit hens welfare [1, 2]. Access to an outdoor range leaves layers exposed to more environmental factors, including weather and soil and environmental micro-organisms, including potential pathogens [3], one of which is the avian influenza virus (AIV) [4]. Layers with access to an outdoor range have an increased risk of low pathogenic AIV introduction [5] via oral ingestion of infected wild bird feces directly or indirectly via an environmental virus reservoir [6, 7]. These environmental factors may also affect the gut microbiota of the layers, and altered cloacal bacterial communities may therefore indicate exposure to the outdoor environment, which may potentially serve as an indicator for the level of biosecurity and exposure to pathogens. Furthermore, understanding the interactions between the gut microbiota in layers and other environmental factors in a commercial setting may yield insights into important drivers of microbiota community composition in layers. This could contribute to better understanding of ways to modulate the microbiota in favour of chicken health and production.

A review by Kers et al. [8] on specific factors that affect the composition of the intestinal microbiota in poultry revealed that in addition to host-related factors like age and breed, environmental factors including housing, litter, feed and climate also affect the composition of intestinal microbiota. Other studies in poultry species have demonstrated that husbandry systems affect the microbiota composition of Pekin ducks [9] and broilers [10]. In layers, access to an outdoor range may result in altered gut microbiota due to exposure to environmental factors including soil, vegetation, natural lighting and rainfall [11]. Additionally, it has been shown that chickens housed in a free-range environment have different microbial community compositions, and increased diversity compared to indoor-housed or caged chickens [11–13]. Xu et al. [12] also reported increased relative abundance of *Bacteroidetes* in free-range chickens, and Hubert et al. [11] reported a higher similarity among the microbiota of free-range chickens compared to caged chickens.

Although in previous studies differences in the microbiota of caged and free-range chickens have been described [9–11], these effects were most likely confounded by the effects of caged compared to non-caged chickens, the breed of the chicken or the age, and did not truly measure the effect of the access to the range. The aim of our study was to determine if there are differences in the composition of the cloacal microbiota in indoor- and outdoor-housed chickens under field conditions. We selected indoor and outdoor flocks based on breed and rearing flock to minimize the effect of other factors than the outdoor range. Cloacal swabs of laying hens from eight commercial layer flocks (four indoor and four outdoor flocks), were analysed to characterize differences in the cloacal microbiota of adult layers with and without access to an outdoor range.

To our knowledge, this is the first report of a cross-sectional study comparing the cloacal microbiota of indoor- and outdoor-housed commercial laying hens. We hypothesized that diversity parameters (i.e., community richness and structure) in outdoor-layers will be higher compared to indoor-layers, because of greater substrate diversity and exposure of the layers to more diverse microbiota in the outdoor environment. Furthermore, we anticipated distinct clustering of the community composition of outdoor-layers compared to indoor-layers due to specific alteration in the community as a result of outdoor exposure.

## Results

We amplified and sequenced the V3-V4 hypervariable region of the 16S rRNA gene. After quality control of all samples with qPCR, samples with low 16S DNA concentration or bad melting curves were removed from further analysis (14 samples in total; 4 negative controls and 10 chicken samples). The final dataset contained 70 samples (7–10 samples per poultry house), with 35 indoor-layer and 35 outdoor-layer samples (Table S2). Each sample was rarefied to 13,154 reads per sample, which was the number of reads in the lowest sample. The final dataset contained a total of 3037 amplicon sequence variants (ASVs).

### Microbial community composition

We evaluated the overall composition of the microbial community in the cloacal samples of all layers. At the phylum level, we observed similarities between the microbiotas of indoor- and outdoor-layers (Fig. S1), and no significant differences in the relative abundances of the ten most abundant phyla were found between indoor- and outdoor-layers. These ten phyla constituted 99.4% ± 1.3 (unless otherwise indicated, results are expressed as mean ± SD) of the community, across all samples. The microbiota in both groups were dominated by *Firmicutes* (54.0% ± 17.3), *Proteobacteria* (15.2% ± 10.2) and *Fusobacteria* (13.6% ± 17.3; Fig. S1). At the genus level, members of the genera *Romboutsia* (22.8% ± 16.0) and *Fusobacterium* (13.5% ± 17.7) were most dominant in both indoor- and outdoor-layers (Fig. S2). *Escherichia/Shigella* were significantly more abundant in outdoor- than indoor-layers (Wilcoxon Rank-Sum test, *p* < 0.005).

### Differences in community structure

**Fig. 1:**
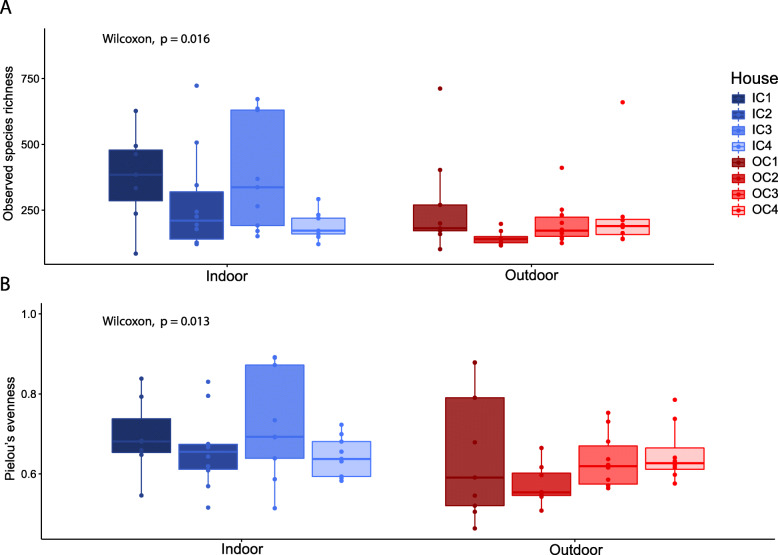
Comparison of observed species richness (**a**) and Pielou’s evenness (**b**) between indoor- (blue) and outdoor-layers (red). Each box contains samples from a single poultry house. Each dot represents an individual chicken. Wilcoxon-Rank-Sum test were performed between indoor- and outdoor-layers. A lower value for Pielou’s evenness indicates less evenness in the microbial community of a sample

**Fig. 2:**
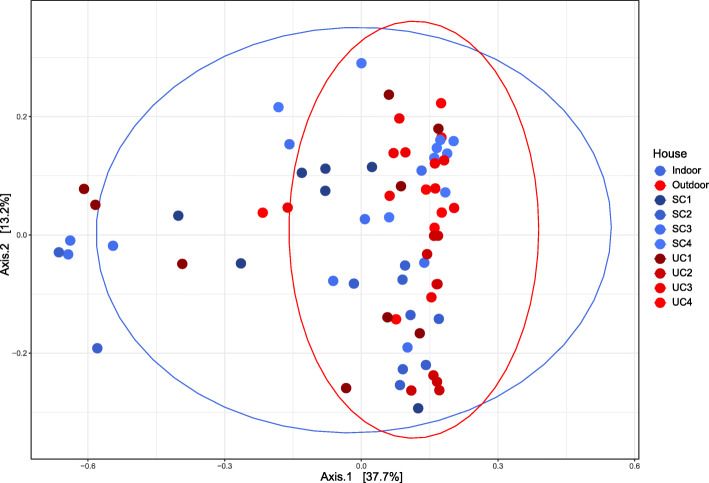
Principal coordinate analysis of Bray Curtis dissimilarity between samples. Color indicates poultry farm and the ellipses the housing type encompassing the 95% CI range of each housing type. Each dot represents an individual sample. Housing type explained 5.6% (R^2^, *adonis, p* = 0.0025) of the variation. Poultry house explained 32.8% (R^2^, *adonis, p* = 0.0001) of the variation

To evaluate the microbial community composition of the layers, we first explored community diversity. Observed species richness (number of ASVs) was significantly higher (Wilcoxon Rank-Sum test, *p* = 0.016) in indoor-layers (302 ± 182 ASVs) compared to outdoor-layers (213 ± 136 ASVs; Fig. 1). Pielou’s evenness was also higher in indoor-layers compared to outdoor-layers (Wilcoxon Rank-Sum test, *p* = 0.013). To evaluate the differences in community structure between indoor- and outdoor-layers, we used a principal coordinate analysis (PCoA) of Bray-Curtis dissimilarities. We found a modest, but significant clustering of microbial communities according to housing type (indoor vs outdoor), explaining 5.6% of the variance in community structure (R^2^; *adonis, p* = 0.0025; Fig. 2). The poultry house where the layers were kept was a much stronger driver of community structure, explaining 32.8% of the variance (R^2^; *adonis p* < 0.001). When ASVs were clustered at the phylum level, no differences between the community composition of different housing types were found (data not shown).

**Fig. 3:**
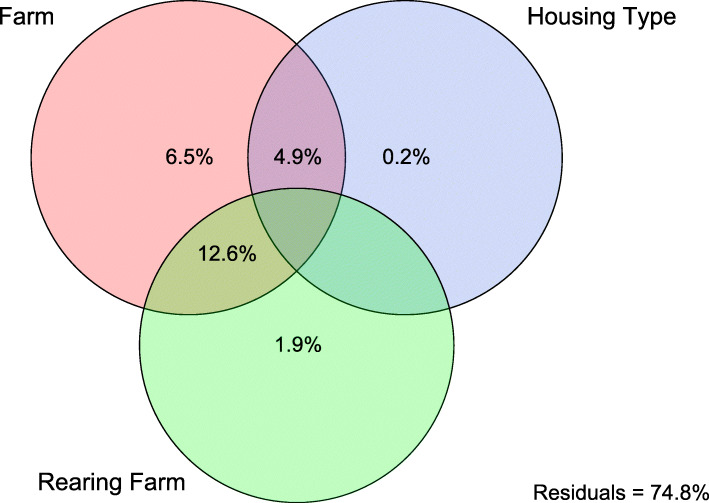
Venn diagram depicting distance-based variation partitioning using Bray-Curtis dissimilarity. The contribution of rearing farm of origin (green, 6.5%), the farm the poultry houses were located in (red), and housing type (indoor or outdoor, blue) to the microbiota composition of layers is shown

To better explain the effect of access to an outdoor range on the variation in microbial community composition, we performed a variation partitioning analysis using Bray-Curtis dissimilarity, with factors housing type (indoor vs outdoor), rearing farm and farm (Fig. 3). Poultry house was excluded from the variation partitioning, as this factor explained most (20.9% R^2^_adj_) of the observed variation and its influence could not be disentangled from other factors due to collinearity with the other factors in our study. Housing type explained the smallest part of the variation (0.2% R^2^_adj_; Fig. 3), whereas the interaction of farm and rearing flock explained most of the variation (12.6% R^2^_adj_). This was followed by farm (6.5% R^2^_adj_), the interaction of housing type and farm (4.9% R^2^_adj_), and rearing farm (1.6% R^2^_adj_).

**Fig. 4:**
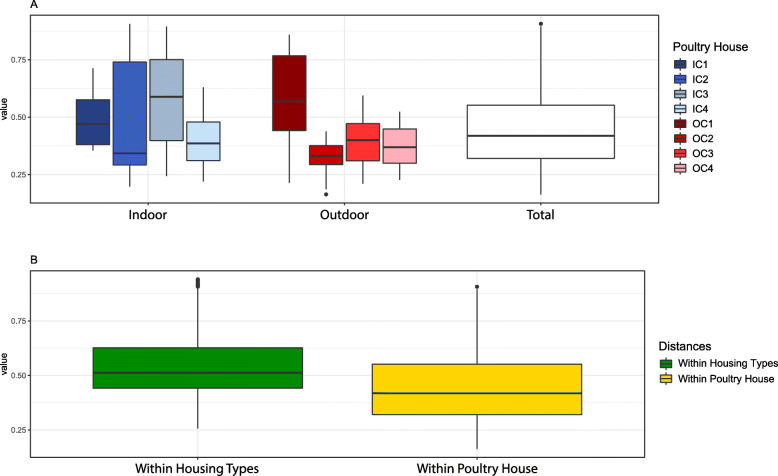
**a** Pairwise Bray-Curtis dissimilarity between the cloacal microbiota of layers from each poultry house. Greater values indicate higher dissimilarity. ‘Total’ contains all possible pairwise comparisons, for reference. Community composition in the indoor poultry houses was more variable than in outdoor poultry houses (Wilcoxon-Rank-Sum test, *p* = 0.03) **b** Bray-Curtis dissimilarities between the cloacal microbiota of layers within a poultry house (Within Poultry House) compared to dissimilarities between cloacal microbiota of layers within a housing type (indoor vs outdoor), excluding within poultry house comparisons (Within Housing Types). The cloacal microbial communities of layers within a poultry house (0.46 ± 0.18, mean ± SD) were more similar to each other than to those within a housing type (mean 0.55 ± 0.15, Wilcoxon-Rank-Sum test, *p* < 0.001)

To examine the variation in microbiota among chickens of the same poultry house, we calculated Bray-Curtis dissimilarities between layers of the same poultry house. Notably, the community composition in the indoor poultry houses was significantly more variable than in outdoor poultry houses (Wilcoxon Rank-Sum test, *p* = 0.03; Fig. 4a). In addition, dissimilarities between outdoor-layers, excluding within-house comparisons, were significantly lower than dissimilarities between indoor-layers (Wilcoxon Rank-Sum test, *p* = 0.002; Fig. S4). Overall, the cloacal microbial communities of chickens within a poultry house were more similar to each other than to those within a housing type (Wilcoxon Rank-Sum test, *p* < 0.001; Fig. 4b).

### Differential abundance of individual taxa

We found five genera which were differentially abundant between indoor- and outdoor-layers (Wilcoxon Rank-Sum test, *p* < 0.005): *Porphyromonas*, *Escherichia/Shigella*, *Sutterella*, *Campylobacter* and *Faecalibacterium* (Fig. S5). Of these, *Escherichia/Shigella* (7.2% contribution to overall Bray-Curtis dissimilarity, *p* = 0.003) and *Porphyromonas* (1.3% contribution to overall Bray-Curtis dissimilarity, *p* = 0.001) contributed most to the dissimilarity between the housing types according to a SIMPER analysis. However, *Porphyromonas* was found in only one outdoor poultry house (OC3) and was absent in samples from all other poultry houses (Fig. S5). *Escherichia/Shigella* was found to have a higher relative abundance in the outdoor flocks, but this increase was specific to poultry houses OC2 and OC3 and not to outdoor flocks OC1 and OC4 (Fig. S5).

*Faecalibacterium* was more abundant in indoor-layers (1.16% ± 2.06) compared to outdoor-layers (0.7% ± 1.86), as well as *Sutterella* (indoor 0.76% ± 1.25; outdoor 0.37% ± 1.09). *Campylobacter* was higher in outdoor-layers (indoor 0.41% ± 0.78; outdoor 1.10% ± 2.55). However, this pattern was specific to individual poultry houses, and none of the genera had a consistently higher or lower relative abundance across all poultry houses of one housing type (Fig. S5).

**Fig. 5:**
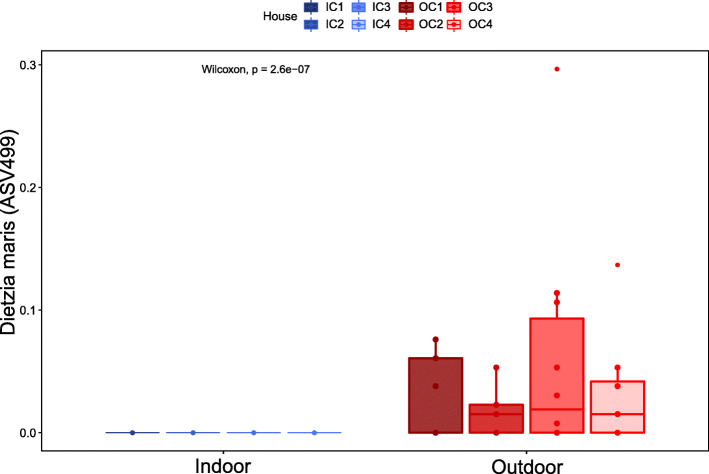
Relative abundance (%) of *Dietzia maris* (ASV499). *Dietzia maris* was present in 57% of the outdoor-layers assessed, and was detected in all outdoor poultry houses. It was not present in any of the indoor-layer samples. Each dot represents an individual chicken. Colors intensity indicates which poultry house the chickens originated from. Wilcoxon-Rank-Sum test was performed between all indoor-layers and all outdoor-layers (*p* < 0.001)

In contrast, we identified a single ASV (Wilcoxon Rank-Sum test, *p* < 0.0001; Fig. 5), which was present in 20 outdoor-layers (57%) across all outdoor poultry houses, with a mean relative abundance of 0.05% ± 0.07% in these 20 layers. This ASV was not present in any of the indoor-layers. A BLAST search [14] of this ASV classified it as a *Dietzia maris* (99.74% identity to strain DSM 43672), which is associated with soil [15].

## Discussion

The evaluation of differences between the cloacal microbiota of indoor- and outdoor-layers in commercial flocks may contribute to an increased understanding of interactions between gut microbiota, housing conditions, and other environmental factors, and help to determine whether the microbiota composition might be used as an indicator of the risk of potential pathogen exposure from the farms’ outdoor environments. Furthermore, understanding the dynamics in microbiota community composition of adult layers in a field setting is relevant, as it may contribute to the insights needed to develop ways to modulate the chickens’ microbiota in favour of health and increased production performance. Although we previously found limited change in the hens fecal microbiota after a single oral inoculation with wild duck feces [16], we hypothesized that continued exposure of laying hens to an outdoor environment would be more likely to result in detectable alterations in the fecal microbiota of outdoor-layers.

**Fig. 6:**
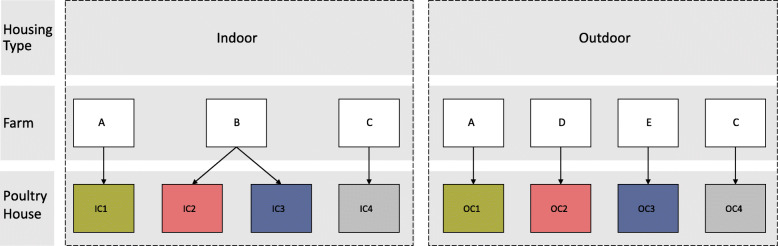
Overview of the study design. Four indoor-laying hen flocks (indoor cross-sectional = IC) and four outdoor flocks (outdoor cross-sectional = OC), each kept in an individual poultry house, were sampled. Chickens from all flocks were of the same breed. Indoor and outdoor flocks that had the same rearing flock of origin were selected, which is indicated with numbers 1, 2, 3 or 4, and colors at the poultry house level. Some poultry houses were situated at the same farm. Hens originating from rearing farm 1 and 4 were placed in the layer farm houses that were situated on the same farm, A and C (Table S1). Farm B housed two flocks (IC2 and IC3) that came from different rearing farms (2 and 3)

In this study we found that access to an outdoor range only explained a small proportion (0.2% R^2^_adj_) of the total variation in the cloacal microbiota of layers. Instead, poultry house was found to be the most important driver of community composition (20.9% R^2^_adj_). When poultry house was excluded from further analysis to more precisely estimate the effect of the outdoor range, the farm where the poultry houses were located (6.5% R^2^_adj_), the rearing farm the chickens originated from (1.9% R^2^_adj_), and the interaction between these two factors (12.6% R^2^_adj_) explained most of the variation. The relatively high R^2^ for this interaction is to be expected, considering the overlap between the factors rearing farm and layer farm in our study design (Fig. 6). We also found that the diversity and evenness in indoor-layers were slightly higher compared to outdoor-layers, suggesting the presence of more dominant species in outdoor-layers. This contrasts with previous studies, which found higher microbial diversity in outdoor-layers [10–13]. Differences in diversity and community composition in previous studies has been related to greater substrate diversity and intake of fibrous feedstuff [12], as well as exposure of the chickens to more abundant microbiota in the outdoor environment [11]. Our findings may deviate from those of previous studies due to several reasons.

In the first place, we selected indoor and outdoor poultry flocks of the same breed (Dekalb White) and based on the rearing farm of origin to minimize variation due to host genetics and rearing conditions. Also, the in-house environment of both indoor- and outdoor-layers was similar in our study. Chickens were housed in cage-free aviary systems, with the same stocking density, feed, minimum number of perches, similar litter etc. This is in contrast with previous research where a comparison was made between either free-range poultry with access to an outdoor range and caged layers [11–13], between fast- and slow-growing broilers [10], or in a semi-experimental set-up [12]. Effects found in these previous studies are likely confounded by the effects of caged vs. non-caged chickens or the breed of the chicken, and not truly measure the effect of the access to the range. Additionally, two studies were performed on either broiler chicks of 42 days of age [10] or indigenous Chinese Dagu chickens, a dual purpose breed which produces both meat and eggs, of only 12 to 18 weeks of age [12]. The microbiota of adult layers develops over time to a stable equilibrium [17], which is less sensitive to external perturbations [16] and hence, may explain the unanticipated limited effect of the outdoor environment in our field study. The timing of access to the range may also be of importance. In the study by Xu et al. [12], Dagu chickens had access to the outdoor range from the beginning of the experiment when the chickens were 6 weeks of age. In contrast, when access to the outdoor range occurred in the last 4 weeks of the cycle in broiler chicks, no change in the richness of the broiler intestinal microbiota was found [18]. The hens in our study were only able to access the range from 19 to 20 weeks of age, after transport from the rearing farm, which means that their microbiotas were almost fully developed and had likely reached a stable equilibrium prior to given access to the range. It is known that a well-developed normal gut microbiota protects the host through creating gastrointestinal resistant environments, which prevent external (pathogenic) bacteria from colonizing the gut [19, 20].

Moreover, it is likely that only a small proportion of the hens in the outdoor flocks of our study visited the outdoor range. Although limited information is available about actual range usage of layers, in the Netherlands it has been estimated that in large flocks (> 10.000 layers) only 3–15% of the hens use the outdoor range at a certain timepoint which is partly dependent on the degree of cover provided by trees or artificial structures in the range [1, 21]. This is supported by Hegelund et al. [22] who found that in commercial layer flocks with access to a range, on average only 9% of the chickens used the range area. In contrast, Gebhardt-Henrich et al. [23] reported that 47–90% of chickens in outdoor flocks were registered in the outdoor range at least once over a period of approximately 3 weeks; the individual hens used the range differently, and many of them did not enter the free-range every day. Furthermore, chickens tended to only use the area immediately outside the hen house [22], which has also been observed in the Netherlands, resulting in trampled vegetation closer to the hen house and hence lower availability of fibrous feedstuff for the hens [24]. The outdoor ranges in the Netherlands in general consist of open fields with some tree coverage and bare soil close to the poultry house [21]. Both the limited use of the outdoor range by the hens, together with the low availability of fibrous feedstuff in the range, may explain why we only found limited effects of the outdoor range on the microbial community composition of layers.

We hypothesized that the microbiota of outdoor-layers would be more variable due to their exposure to the outdoor environment and the fact that not all layers use the outdoor range. However, we found more variability in microbiota of indoor-layers compared to the outdoor-layers. Previous research has shown that microbiota of free-range layers contained a greater variability of bacterial species compared to caged layers [25, 26]. The greater variability in the bacterial community composition of the chickens with only indoor housing in our study could be a result of the spatial distribution of the hens within the poultry house. In all flocks in this study, compartments were present in the indoor area of both the flocks with indoor housing and those with access to the outdoor range. This is according to Dutch regulations, that stipulate that poultry houses need to be divided into compartments which contain no more than 6000 hens [27]. Although outdoor poultry houses also have these compartments, layers are able to move freely between these compartments because they have access to the range and can enter another compartment from the outdoor range. This means that the outdoor-layers are more evenly distributed across the poultry house, whereas strictly indoor-layers stay in the same separate compartment within the poultry house all the time. Consequently, this increased level of compartmentalization in indoor-layers can cause a so called cage-effect, which has been reported in several animal studies [26, 28], and could explain the higher variation between layers from indoor houses. Unfortunately, we were not able to adequately measure this effect, because we did not take the compartmentalization into account when sampling the flocks. In future studies where different housing types are being compared with regard to microbiota composition, potential impact of differences in compartmentalisation should be taken into account in the study design.

In order to sample commercial flocks, we opted for cloacal swabs, which served as a rapid and accurate sampling methodology that did not entail sacrificing the birds. The fecal microbiota of chickens are qualitatively similar to the cecal microbiota [29], but more variable [30]. While our sampling technique may explain why we found a high degree of variation between individual chickens, it does not explain the differences in variability in the microbiota of layers of different housing types, as the same sampling technique was used across the study. We found one specific ASV, *Dietzia maris,* that was only found in outdoor-layers and is related to soil [15]. However, this was a lowly abundant taxon, and we did not detect differences between indoor- and outdoor-layers when we looked at the all ASVs in the genus *Dietzia* jointly. Moreover, no other genera were found to be differentially abundant across all indoor and outdoor poultry houses. This suggests that although the chickens can pick up some specific taxa from the range, access to an outdoor range does not cause a distinct shift in the microbial community of layers. Therefore, we cannot use community-wide microbiota assessments as a measure for biosecurity or exposure to pathogens from the outdoor environment of a farm.

This study furthermore emphasises the importance of the environment of the poultry house, and the likely influence of daily management on the fecal microbiota, which was also found in broilers [31] and several murine models [32]. In the study by Kers et al. [31], broiler chicks were raised in different housing environments, and were given two diets. These feed interventions alone explained 10% (R^2^) of the variation in microbiota composition between the broilers, whereas housing condition alone explained 28% (R^2^). The effect of the poultry house environment explained a similar amount of variation in our study. Future research should aim at better understanding the interactions between the gut microbiota in layers and environmental factors at the level of the poultry house over time. This may shed light on important drivers of microbiota community composition in commercial layers and could contribute to better understanding of ways to modulate the microbiota in favour of chicken health and production.

## Conclusions

This cross-sectional field study shows that exposure to an outdoor environment is responsible for a relatively small proportion of the community variation in the microbiota of layers. We did not detect unique patterns in the community composition of outdoor-layers compared to indoor-layers or detect specific microbiota that could be related to contact with an environment contaminated by wild birds. Overall, measuring differences in cloacal microbiota of layers as an indicator for the level of exposure to potential pathogens and biosecurity seems of limited practical use. To be able to gain more insight into environmental drivers of the gut microbiota that may be associated with pathogen exposure, and hence performance, future research should aim at investigating community composition of commercial layer flocks over time.

## Material and methods

### Study design and sample collection

Eight commercial flocks of laying hens (Dekalb White) were selected for cloacal sampling: four layer flocks with access to an outdoor range (outdoor flocks) and four flocks without access to an outdoor range (indoor flocks). To minimize potential variation in the microbiota composition due to rearing and other environmental factors, outdoor and indoor flocks were selected based on the rearing farm of origin as well as on age (Fig. 6, Table S1). All flocks consisted of layers between 27 and 40 weeks of age, which are assumed to have matured to a stable gut microbiota composition [17]. Flocks from the same rearing farm, were of the same age, and were sampled within the same week. All flocks were sampled within the same month (October 2017) to avoid short term weather and seasonal effects. The sampled flocks were kept in separate poultry houses, which were located on five different poultry farms: two indoor and outdoor flocks were kept in poultry houses at the same farm, two indoor flocks were located in poultry houses at the same farm, and two outdoor flocks were housed at two separate farms (Fig. 6). All flocks were healthy at the time of sampling, and had not been treated with antibiotics on the layer farm. Both indoor and outdoor-layer flocks were kept in a cage-free aviary system with a maximum stocking density of nine chickens per m^2^, with one flock per poultry house [27]. The laying hens of the outdoor flocks had access to an outdoor range during the day with at least 4 m^2^ per hen according to standards of the Dutch quality assurance scheme, i.e. the Integrated Chain Control program, ‘IKB Egg’ [33]. The chickens had access to the outdoor range for 8 h a day on average [21]. Outdoor ranges were mostly open grass field with some trees, and bare soil directly around the poultry house and drainage systems to prevent formation of rain puddles [21]. Outdoor ranges were all fenced off with chicken wire.

In each flock, two cloacal dry swabs were collected from 50 laying hens. The poultry houses contained several subsections, and an equal number of birds was randomly selected from each subsection of each flock. Samples were placed on ice immediately after collection and stored at − 80 °C within 5 h after collection.

### DNA extraction and 16S rRNA gene amplicon sequencing

Per flock, one cloacal swab of a selection of ten chickens, was chosen for further analysis. Swabs of chickens were selected based on equal distribution across the farm and visual assessment of the swab to ensure that sufficient fecal material was present for DNA extraction. DNA extraction was performed according to the protocol in Schreuder et al. [16]. In each DNA isolation round a negative control sample containing PBS was added to identify possible contamination from reagents. Following extraction, the DNA extracts were quantified with Invitrogen™ Qubit™ 3.0 Fluorometer and stored at − 20 °C for further processing. The V3–V4 region of the 16S rRNA gene was amplified in a PCR with the primers CVI_V3-forw CCTACGGGAGGCAGCAG and CVI_V4-rev GGACTACHVGGGTWTCT. The following amplification conditions were used as previously described [16]: step 1: 98 °C for 2 min, step 2: 98 °C for 10 s, step 3: 55 °C for 30 s, and step 4: 72 °C for 10 s, step 5: 72 °C for 7 min. Steps 2 to 4 were repeated 25 times. Negative controls were included at each amplification round to confirm sterility of PCR reagents. PCR products were checked with gel electrophoresis, and PE300 sequencing was performed using a MiSeq sequencer (Illumina Inc., San Diego, CA). An additional 16S rRNA gene qPCR was performed on the DNA samples, to quantify the amount of 16S rRNA gene DNA and identify samples of poor quality (Table S2). An additional two samples did not have good quality melting curves, and these samples were discarded from further analyses. The qPCR consisted of 40 cycles with the same primers and protocol as for the PCR.

### Processing of sequencing data

All sequence processing was performed in R 3.5.1 [34]. The sequence reads were filtered, primer-trimmed (35 nucleotides), dereplicated, chimera-checked, and merged using the *dada2* package [35] using standard parameters (TruncLength = 240,210, MinOverlap = 1 and maxEE = (2,2)). Reads were assigned with the SILVA v.132 classifier [36]. Negative controls from the DNA extraction did not contain any sequences above detection level and were discarded (*n* = 4). Some of the samples (*n* = 10) contained very low 16S DNA concentration after the qPCR or gave poor quality melting curves, and were discarded after sequencing. The final dataset contained 70 samples. In the final dataset the number of samples per poultry house ranged between seven and ten samples, with 35 indoor-layer and 35 outdoor-layer samples (Table S2).

### Statistical analyses

All downstream analyses were performed in R (version 3.5.1) with the phyloseq [37] and vegan [38] R packages. We measured diversity as the number of observed ASVs in a sample, and evaluated species evenness within samples with Pielou’s index J [39] at the species level. Bray-Curtis dissimilarity measure was used to evaluate differences in community structure between the layers on Hellinger transformed ASV abundances in phyloseq [40], and selected ASVs with a total sum value of greater than 1. Factors that were included in further analysis were housing type (indoor- and outdoor-layers), poultry house (stable in which flocks were housed), rearing flock (rearing flock where layers from a flock originated from), and farm (farm where poultry houses were based, i.e. some farms had multiple houses, Fig. 6). Feed was not included in the analyses as this could not be disentangled from the effect of the poultry house. Differences between the microbiota composition of layers were examined for each factor using the *adonis* function on Bray-Curtis dissimilarity [41]. To further assess the contribution of each factor to the observed variation in the microbiota composition, we performed distance-based (Bray-Curtis) redundancy analysis [41]. A model with housing type, and poultry house (Fig. S3) was most parsimonious, explaining 31.8% of the variation. As poultry house explained most of the variation in the microbiota composition, we further disentangled the contribution of the factors farm, rearing farm, and housing type with distance-based variation partitioning, leaving poultry house as a factor out of the model [42]. To test how well samples from individual layers within one poultry house represented the microbiota of that house, we calculated community Bray-Curtis dissimilarity between layers within each poultry house. Additionally, we calculated community Bray-Curtis dissimilarity between layers of the same housing type, excluding the comparisons between layers of the same poultry house, to evaluate how well samples from individual layers of one housing type represented the microbiota of that housing type.

We used two approaches, Wilcoxon Rank-Sum tests and DESeq2 [43], to check for differences in relative abundances of the ten most abundant phyla, 0.5% most abundant genera and 0.01% most abundant ASVs. We present only the result of the Wilcoxon Rank-Sum test, as this non-parametric test is most suitable for high variability between samples, and only this approach identified taxa which were consistently higher in one condition. Taxa for which Wilcoxon Rank-Sum test resulted in *p* < 0.01 were selected for further analyses. We used a SIMPER analysis to identify which of the genera contributed most to the beta diversity [44]. With Wilcoxon Rank-Sum test we identified if specific ASV were consistently increased or decreased in either of the two housing types. The figures from ggplot2 and ggpubr were further refined in Adobe Illustrator CC (version 21.0.2.)

## Supplementary information

**Additional file 1: Table S1.** Overview of flocks that were sampled for the cross-sectional study. Rearing farm is the outdoor and indoor flock that were paired based on rearing farm of origin.

**Additional file 2: Table S2.** Sample list.

**Additional file 3: Figure S1.** Relative abundances of the ten most abundant phyla within all indoor- and outdoor-layers. Overall, these phyla constituted 99.4% ± 1.3 (mean ± SD) of the community across all samples

**Additional file 4: Figure S2.** Relative abundance of the 15 most abundant genera across all samples faceted per housing type. Only *Escherichia/Shigella* had a significant difference between indoor- and outdoor-layers (*p* < 0.005, Wilcoxon-Rank-Sum test).

**Additional file 5: Figure S3.** Distance-based redundancy analysis (RDA) using the following model: y = Housing Type + Housing Type:Poultry House. This parsimonious model explained 31.8% of the variation (R^2^). Circles represent individual chickens. Colors indicate which poultry house the chickens originated from.

**Additional file 6: Figure S4.** Pairwise Bray-Curtis dissimilarities between the cloacal microbiota of layers from each housing type, excluding within poultry house comparisons. Greater values indicate higher dissimilarity. The ‘total’ box contains all possible pairwise comparisons, for reference. Community composition between indoor-layers was more variable than between outdoor-layers (Wilcoxon-Rank-Sum test, *p* = 0.002)

**Additional file 7: Figure S5.** Relative abundances (%) of the five genera that were significantly different between indoor- and outdoor-layers on Wilcoxon-Rank-Sum test (*p* < 0.01). Dots represent individual chickens. Colors indicate which poultry house the chickens originated from. Wilcoxon-Rank-Sum test were performed between all indoor-layers and all outdoor-layers.

## Data Availability

Availability of data and materials Raw sequence data were submitted into the Sequence Read Archive (SRA) at the NCBI under accession number PRJNA613267.

## Abbreviations

16S rRNA: 16 Svedberg ribosomal ribonucleic acid
AIV: Avian influenza virus
ASV: Amplicon sequence variant
IC: Indoor Cross-sectional
OC: Outdoor Cross-sectional
PCR: Polymerase chain reaction
qPCR: Quantitative polymerase chain reaction
h: hour
C: Celsius
DESeq: Differential expression analysis for sequence count data
SIMPER: Similarity percentages
PCoA: Principal coordinate analysis
BLAST: Basic local alignment search tool
RDA: Redundancy analysis
