# Supplementary material for: An observational field study of the cloacal microbiota in adult laying hens with and without access to an outdoor range
Source: Anim Microbiome. 2020 Aug 8;2:28. doi: 10.1186/s42523-020-00044-6 (PMC7807755; doi:10.1186/s42523-020-00044-6)
Supplement: Supplementary file 1 — Additional file 1: Table S1. Overview of flocks that were sampled for the cross-sectional study. Rearing farm is the outdoor and indoor flock that were paired based on rearing farm of origin. [file 42523_2020_44_MOESM1_ESM.docx]

Additional files

Additional file 1: **Table S1.** Overview of flocks that were sampled for the cross-sectional study. Rearing farm is the outdoor and indoor flock that were paired based on rearing farm of origin.

| Rearing farm | Flock | Type | Age (weeks) | Farm | Sampling date | # of samples in final dataset |
| --- | --- | --- | --- | --- | --- | --- |
| 1 | OC1^a^ | Outdoor | 27 | A | 23-10-2017 | 9 |
| 1 | IC1^a^ | Indoor | 27 | A | 23-10-2017 | 7 |
| 2 | OC2 | Outdoor | 29 | D | 25-10-2017 | 8 |
| 2 | IC2^b^ | Indoor | 29 | B | 18-10-2017 | 10 |
| 3 | OC3 | Outdoor | 35 | E | 17-10-2017 | 10 |
| 3 | IC3^b^ | Indoor | 35 | B | 18-10-2017 | 10 |
| 4 | OC4^c^ | Outdoor | 40 | C | 10-10-2017 | 8 |
| 4 | IC4^c^ | Indoor | 40 | C | 10-10-2017 | 9 |

^a^ Flocks situated in different poultry houses on same farm

^b^ Flocks situated in different poultry houses on same farm

^c^ Flocks situated in different poultry houses on same farm
