# Supplementary figures and images for: An observational field study of the cloacal microbiota in adult laying hens with and without access to an outdoor range
Source: Anim Microbiome. 2020 Aug 8;2:28. doi: 10.1186/s42523-020-00044-6 (PMC7807755; doi:10.1186/s42523-020-00044-6)

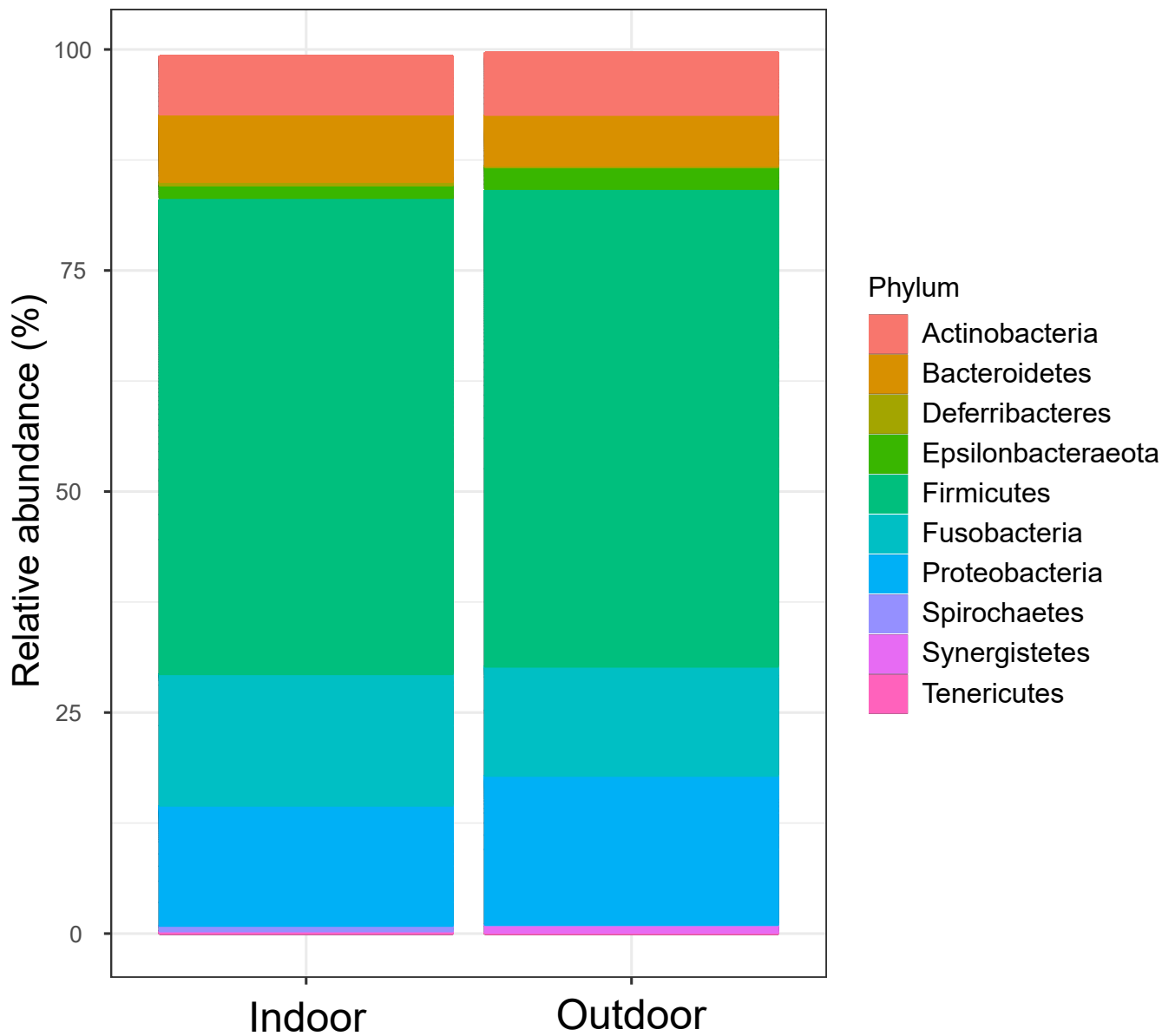

Supplement: Supplementary file 3 — Additional file 3: Figure S1. Relative abundances of the ten most abundant phyla within all indoor- and outdoor-layers. Overall, these phyla constituted 99.4% ± 1.3 (mean ± SD) of the community across all samples [file 42523_2020_44_MOESM3_ESM.pdf]

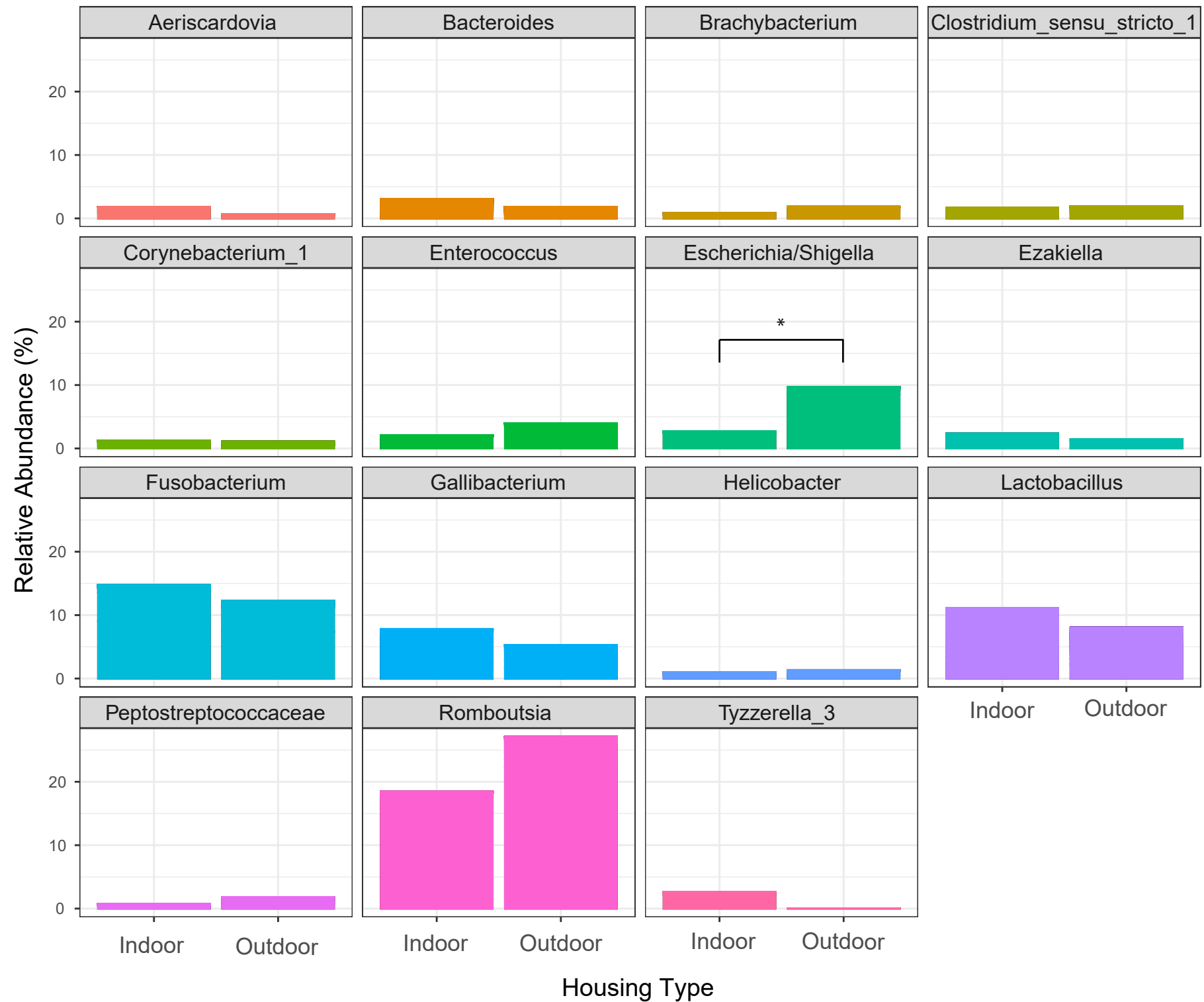

Supplement: Supplementary file 4 — Additional file 4: Figure S2. Relative abundance of the 15 most abundant genera across all samples faceted per housing type. Only Escherichia/Shigella had a significant difference between indoor- and outdoor-layers (p < 0.005, Wilcoxon-Rank-Sum test). [file 42523_2020_44_MOESM4_ESM.pdf]

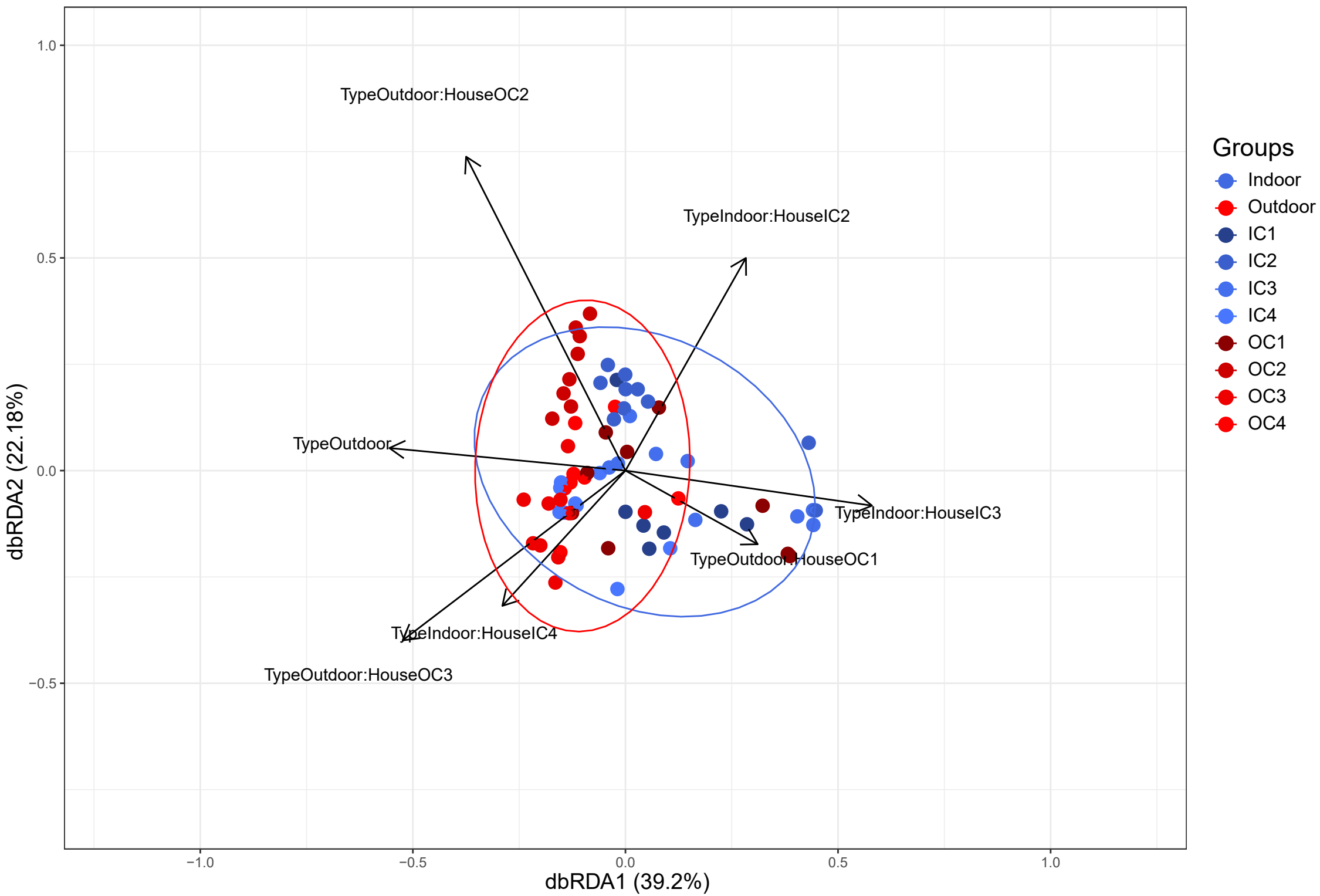

Supplement: Supplementary file 5 — Additional file 5: Figure S3. Distance-based redundancy analysis (RDA) using the following model: y = Housing Type + Housing Type:Poultry House. This parsimonious model explained 31.8% of the variation (R2). Circles represent individual chickens. Colors indicate which poultry house the chickens originated from. [file 42523_2020_44_MOESM5_ESM.pdf]

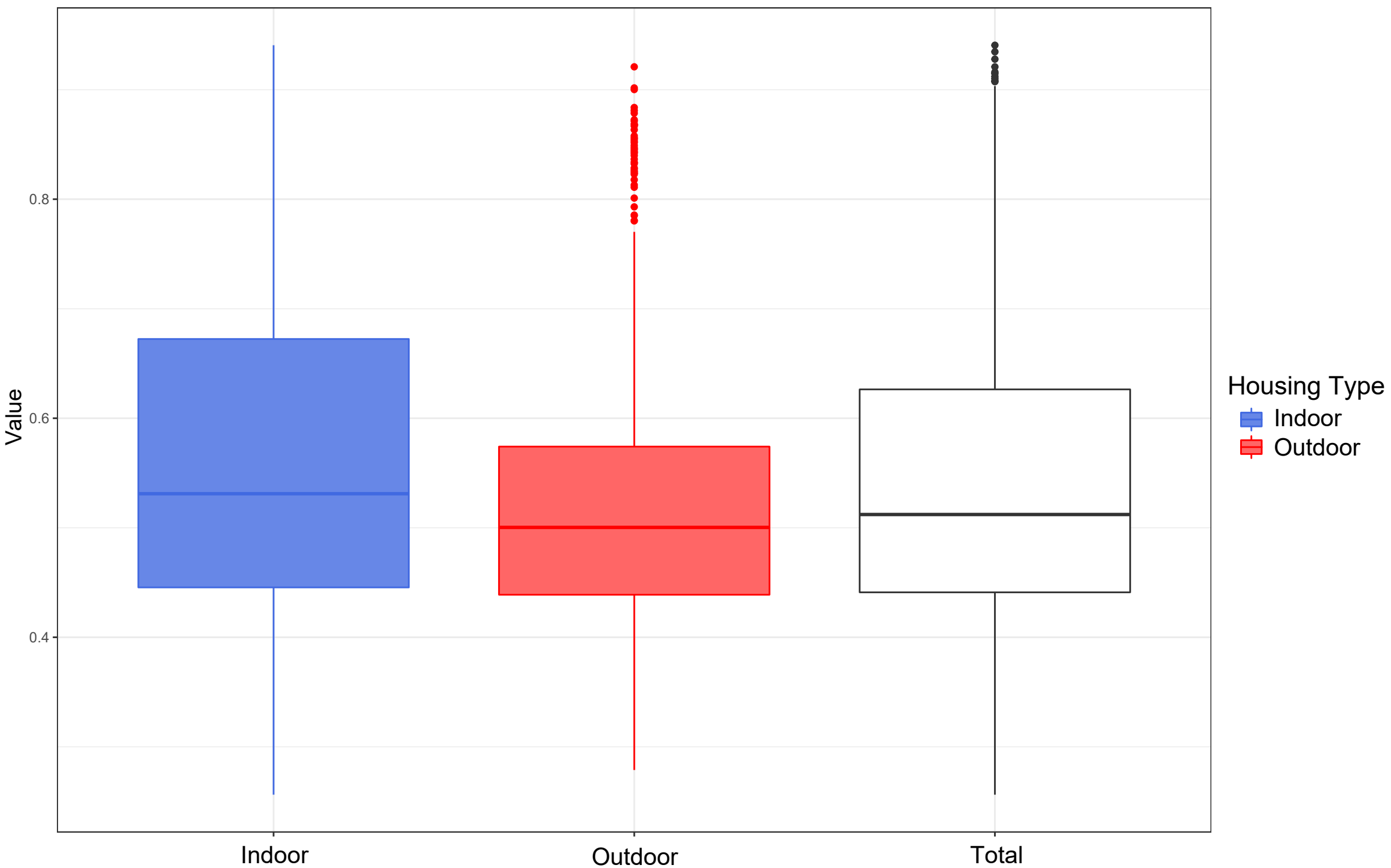

Supplement: Supplementary file 6 — Additional file 6: Figure S4. Pairwise Bray-Curtis dissimilarities between the cloacal microbiota of layers from each housing type, excluding within poultry house comparisons. Greater values indicate higher dissimilarity. The ‘total’ box contains all possible pairwise comparisons, for reference. Community composition between indoor-layers was more variable than between outdoor-layers (Wilcoxon-Rank-Sum test, p = 0.002) [file 42523_2020_44_MOESM6_ESM.pdf]

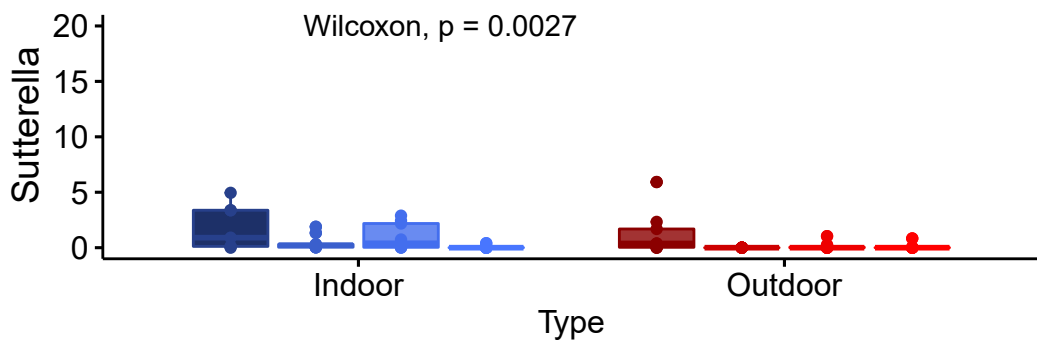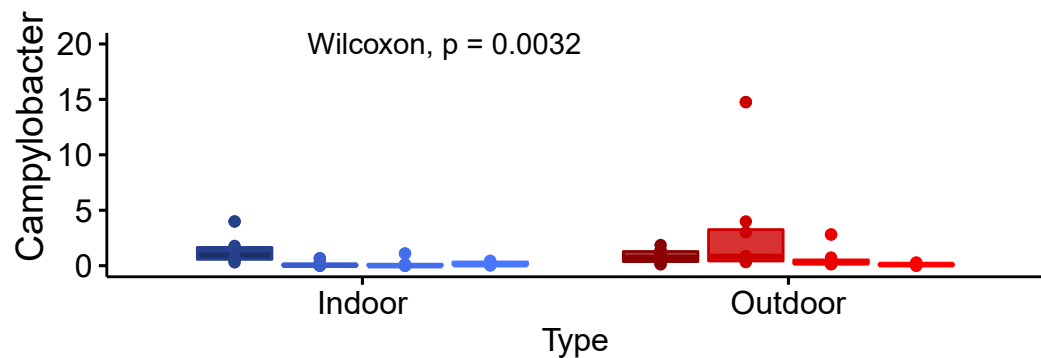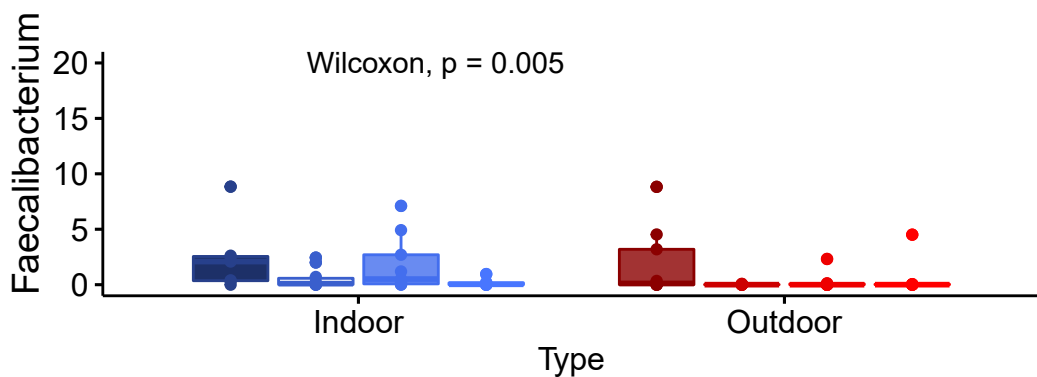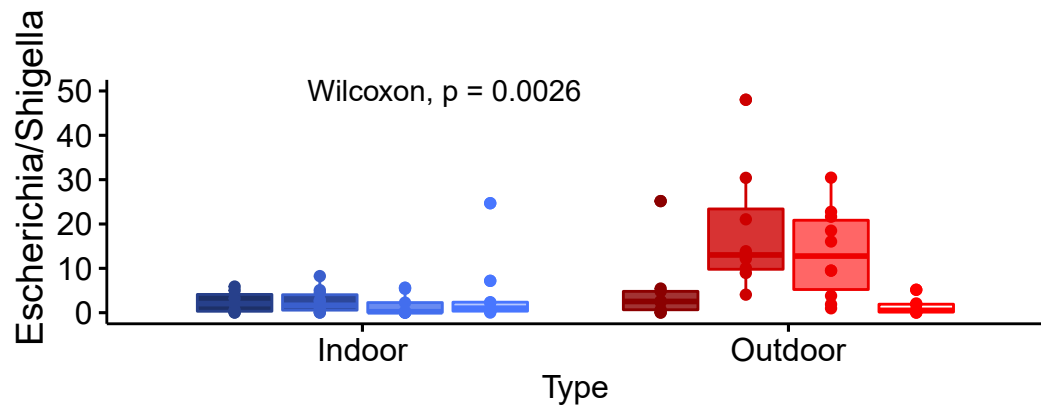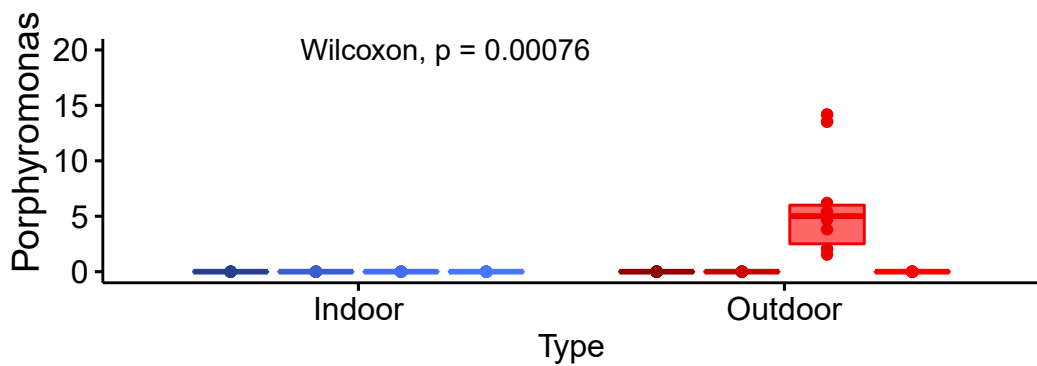

House

|     |     |     |     |
|-----|-----|-----|-----|
| IC1 | IC3 | OC1 | OC3 |
| iC2 | IC4 | OC2 | OC4 |

Supplement: Supplementary file 7 — Additional file 7: Figure S5. Relative abundances (%) of the five genera that were significantly different between indoor- and outdoor-layers on Wilcoxon-Rank-Sum test (p < 0.01). Dots represent individual chickens. Colors indicate which poultry house the chickens originated from. Wilcoxon-Rank-Sum test were performed between all indoor-layers and all outdoor-layers. [file 42523_2020_44_MOESM7_ESM.pdf]
